# *CDKN2A*/*p16* Exon 2 Hypermethylation in Lung Squamous Cell Carcinoma Associated with Interstitial and Emphysematous Lung Diseases: A Comparative Analysis of Tumor, Adjacent and Distant Lung Tissues

**DOI:** 10.3390/curroncol33040187

**Published:** 2026-03-27

**Authors:** Keita Miyakawa, Kyohei Oyama, Jiayao Liu, Naoko Akiyama, Akira Sakata, Manami Hayashi, Yuki Kamikokura, Naoko Aoki, Sayaka Yuzawa, Shin Ichihara, Takaaki Sasaki, Masahiro Kitada, Yusuke Mizukami, Mishie Tanino

**Affiliations:** 1 Department of Diagnostic Pathology, Asahikawa Medical University Hospital, Asahikawa 078-8510, Japan; miyakawa@hirosaki-u.ac.jp (K.M.);; 2 Department of Bioscience and Laboratory Medicine, Hirosaki University Graduate School of Health Sciences, Hirosaki 036-8564, Japan; 3 Department of Cardiac Surgery, Asahikawa Medical University, Asahikawa 078-8510, Japan; 4 Division of Respiratory Medicine and Neurology, Department of Internal Medicine, Asahikawa Medical University, Asahikawa 078-8510, Japan; 5 Department of Thoracic Surgery and Breast Surgery, Asahikawa Medical University Hospital, Asahikawa 078-8510, Japan; 6 Division of Gastroenterology, Department of Medicine, Asahikawa Medical University, Asahikawa 078-8510, Japan; 7 Center for Intractable Diseases and ImmunoGenomics, National Institutes of Biomedical Innovation, Health and Nutrition, Osaka 567-0085, Japan

**Keywords:** lung squamous cell carcinoma, *p16* (*CDKN2A*) exon 2, *CDH13*, *RASSF1A*, DNA methylation, field cancerization, idiopathic pulmonary fibrosis, pulmonary emphysema, smoking-related interstitial fibrosis

## Abstract

Lung squamous cell carcinoma (LUSC) tends to arise in the setting of interstitial or emphysematous lung diseases, including idiopathic pulmonary fibrosis (IPF), pulmonary emphysema (PE), and smoking-related interstitial fibrosis (SRIF), where field cancerization may extend. DNA methylation of promoter regions of *p16*, *CDH13*, and *RASSF1A* and *p16* exon 2 was assessed by methylation-specific PCR. Tumor, adjacent (<3 cm), and distant (≥3 cm) lung tissues were obtained from 25 patients with LUSC (IPF, *n* = 7; PE, *n* = 8; SRIF, *n* = 10). *p16* exon 2 methylation was significantly higher in tumors than in non-tumorous tissues in PE and SRIF cases. In contrast, IPF cases showed *p16* exon 2 hypermethylation also in distant tissues. Across tumor samples, *p16* promoter hypermethylation was frequently observed in stage II or higher. *p16* expression in tumors was generally reduced in IPF and PE cases, compared with SRIF cases. No consistent methylation or expression patterns were observed for *CDH13* or *RASSF1A*. *p16*-associated molecular alterations exhibited disease- and stage-related differences, suggesting heterogeneity in LUSC carcinogenesis. These findings indicate a broader epigenetic field effect, as reflected by *p16* exon 2, in IPF-associated LUSC and suggest that complex, elusive mechanisms underlying *p16* aberrations may contribute to this phenomenon.

## Simple Summary

Lung squamous cell carcinoma (LUSC) frequently develops in patients with chronic lung diseases, including idiopathic pulmonary fibrosis (IPF), pulmonary emphysema, and smoking-related interstitial fibrosis (SRIF); however, the underlying carcinogenetic mechanisms remain unclear. We investigated selected molecular alterations in tumor and non-tumorous lung tissues from patients with LUSC arising in these underlying diseases. Methylation-specific PCR revealed frequent *p16* exon 2 methylation in tumor tissues across all cases. Notably, *p16* exon 2 methylation was also detected in distant non-tumor lung tissue from patients with IPF, a pattern not observed for the promoter of *p16*, *CDH13*, or *RASSF1A*. Furthermore, p16 protein expression was lower in IPF- or PE-associated LUSC than in SRIF-associated LUSC. These results suggest that the carcinogenetic processes underlying LUSC may vary according to the type of pre-existing lung disease. Elucidating these disease-specific molecular pathways may contribute to improved detection and therapeutic strategies for LUSC in patients with chronic lung disorders.

## 1. Introduction

Lung cancer remains the leading cause of cancer-related mortality worldwide. Despite recent advances in therapy, prognosis remains poor, particularly in patients with fibrotic or smoking-related lung disease [1,2]. Accumulating evidence suggests that lung carcinogenesis may occur within a context of field cancerization (FC), in which molecular abnormalities extend beyond the tumor itself and affect broad areas of the lung epithelium [3]. FC has been documented in lung adenocarcinoma (LUAC) [4,5] and non-small cell lung cancer (NSCLC) [6], with widespread molecular alterations that predispose the lungs to malignant transformation. Consequently, pulmonary diseases associated with an increased risk of lung cancer may harbor extensive molecular aberrations even in histologically non-tumorous tissue.

Idiopathic pulmonary fibrosis (IPF) [1,2,7,8,9], and pulmonary emphysema (PE) [10,11] are strongly associated with oncogenic processes and precancerous field expansion in lung squamous cell carcinoma (LUSC). Cigarette smoke-induced injury promotes cellular senescence, which may lead either to impaired tissue repair, as typically observed in PE [12], or to progressive fibrosis through the accumulation of senescent fibroblasts and extracellular matrix (ECM), as seen in IPF [8,12,13] and smoking-related interstitial fibrosis (SRIF) [14,15]. IPF is characterized by subpleural-predominant fibrosis, a progressive clinical course, poor prognosis, and a markedly increased risk of lung cancer [1,7,8]. In contrast, SRIF exhibits centrilobular-predominant fibrosis and a generally non-progressive course, with no clear evidence of a similarly poor prognosis or elevated lung cancer risk [14,15,16]. Previous studies have shown that, in IPF, lung cancer frequently arises at the interface between fibrotic and relatively preserved lung parenchyma [1,2], whereas in emphysema, tumors tend to develop in regions with more severe emphysematous destruction [17]. In contrast, SRIF-associated tumors do not demonstrate a consistent anatomical predilection [2]. These differences suggest that persistent epithelial injury and abnormal repair may contribute to heterogeneity in FC across underlying lung diseases and may be reflected in disease-specific DNA methylation patterns, including *p16* methylation. Indeed, LUSC associated with IPF has been reported to exhibit distinct methylation profiles compared with LUSC without IPF [1], supporting the notion that epigenetic alterations provide molecular evidence of FC. Based on these observations, we hypothesized that the extent and spatial distribution of FC-related epigenetic alterations differ among LUSC arising in IPF, PE, and SRIF.

Among studies investigating DNA methylation in NSCLC, hypermethylation of the promoters of *p16* [18,19,20,21], *CDH13* [18,19,20,22], and *RASSF1A* [18,19,20,22] has been frequently reported as diagnostic or prognostic markers. In addition, methylation of *p16* exon 2 has been observed in lung cancer [22,23] and its precursor lesions [22]. Moreover, the promoter regions of *p16* [20,24,25], *CDH13* [26], and *RASSF1A* [26] demonstrate significantly increased methylation with cumulative smoking exposure. Therefore, when evaluating FC across IPF, PE, and SRIF, smoking-related hypermethylation may mask disease-specific signals; thus, *p16* exon 2 may represent an important marker. Promoter methylation of *p16* is generally considered an early event in carcinogenesis because it directly suppresses gene transcription [21,24]. In contrast, methylation within the gene body, such as exon 2, has been suggested to be associated with tumor progression rather than transcriptional silencing [22,23,27].

However, the causal roles and mechanisms by which these epigenetic alterations contribute to lung carcinogenesis remain incompletely understood. *p16* functions as a key regulator of the cell cycle and a marker of cellular senescence, and reduced p16 expression has been reported in NSCLC [28] and LUSC [29,30], correlating with unfavorable clinical outcomes [28,29,30]. *CDH13* encodes as a tumor-suppressor cadherin involved in inhibition of proliferation, migration, and invasion, as well as regulation of epithelial–mesenchymal transition (EMT)-related pathways [31]. *RASSF1A* is a well-established tumor suppressor that is frequently silenced by promoter hypermethylation in NSCLC, resulting in enhanced EMT, motility, invasiveness, and metastatic potential [32], and in LUAC has been linked to collagen-mediated ECM stiffening and increased tumor dissemination [33]. Both IPF and PE are strongly associated with cellular senescence [12,13,34], and EMT plays a crucial role in the development and progression of lung cancers arising in fibrotic lungs [35]. In IPF, basal cell-like epithelial cells expressing *p16* and EMT-related genes localize to the epithelial layer covering fibroblastic foci and are absent in normal lungs [30]. In SRIF, fibroblastic foci contain an increased number of cells with exhibiting EMT-like immunohistochemical features [36]. *p16* [13,28,29,30,34,37,38], *CDH13* [31], and *RASSF1A* [32,33] are thus implicated in biological processes common to cancer, IPF, PE, and SRIF, including EMT [35,36,39], fibrosis and ECM deposition [8,12,13,35], and cellular senescence [8,12,40]. Collectively, these findings indicate that these genes may play pivotal roles in FC and carcinogenesis in LUSC arising from fibrotic and smoking-related lung diseases.

Based on these considerations, we hypothesized that epigenetic alterations of *p16*, *CDH13*, and *RASSF1A* may differ according to the underlying fibrotic or smoking-related lung disease and may contribute to local lung carcinogenesis. To test this hypothesis, we analyzed methylation status and protein expression in LUSC arising in the background of IPF, SRIF, and PE and compared background lung tissues from sites where carcinoma arose with those from areas where carcinoma did not develop.

## 2. Materials and Methods

### 2.1. Patients

Among 750 patients who underwent surgical resection for primary lung cancer at Asahikawa Medical University Hospital between 2018 and 2023, 149 were diagnosed with primary LUSC. The diagnoses of LUSC, IPF, SRIF, and PE were established based on clinical, radiological, and pathological findings by at least two board-certified pathologists. The diagnosis of IPF was made according to the current international guidelines. Background lung tissues were histologically evaluated to confirm disease-specific pathological features; cases with concomitant connective tissue disease or infection were excluded, and PE was defined by emphysematous change without other background lesions. Patients who received neoadjuvant therapy or lacked adequately sized resection specimens containing both tumor and tumor-distant background lung tissue for sufficient DNA extraction were also excluded. Based on these criteria, 25 patients were included in the study (IPF, *n* = 7; PE, *n* = 8; and SRIF, *n* = 10). “IPF cases” were defined as LUSC arising in lungs with IPF; the same definition was applied to PE and SRIF. Smoking history was expressed in pack-years. The clinicopathological characteristics of the participants are summarized in Table 1, with N0 and M0 including clinically confirmed cases. The study was approved by the ethics committee of Asahikawa Medical University (approval no. 21055) and was conducted in accordance with the principles of the Declaration of Helsinki.

### 2.2. Tissue Samples

**Figure 1:**
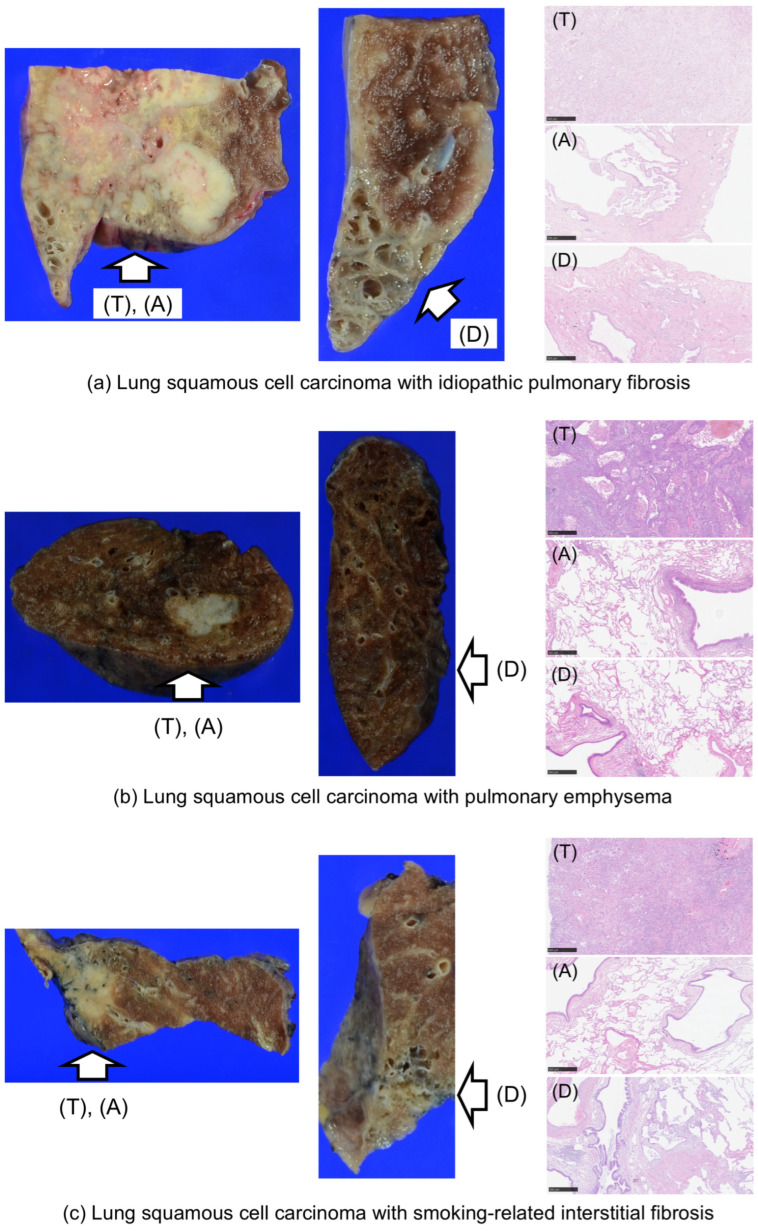
Schematic representation of lung tissue sampling regions: tumor, adjacent, and distant non-tumor areas. (**a**) Shows representative examples of the methylation analysis regions from IPF samples, including T (tumor), A (tumor-adjacent lung tissue within 3 cm of the tumor), and D (tumor-distant lung tissue located ≥3 cm from the tumor). Corresponding representative regions for PE and SRIF are shown in (**b**) and (**c**), respectively. All histological images correspond to ×5 objective magnification fields (Scale bars = 500 µm). IPF, idiopathic pulmonary fibrosis; PE, pulmonary emphysema; SRIF, smoking-related interstitial fibrosis.

Following surgical resection, lung tissues were fixed in 10% neutral-buffered formalin for 24–72 h and embedded in paraffin to prepare formalin-fixed paraffin-embedded (FFPE) blocks. For methylation analysis, three types of samples were obtained from the FFPE blocks via macro-dissection (Figure 1): tumor tissues (T), adjacent background lung tissues within 3 cm of the tumor (A), and distant background lung tissues located ≥3 cm from the tumor (D). Tumor specimens were selected to ensure a high proportion of tumor cells. Areas showing histologic features representative of the respective background lung diseases (e.g., IPF, PE, or SRIF) were preferentially sampled. Immunohistochemical staining was performed on the same FFPE blocks used to obtain tumor samples (T) for DNA methylation analysis.

### 2.3. DNA Extraction and Methylation Analysis

DNA was extracted from tissue samples using the QIAamp DNA FFPE Advanced UNG Kit (QIAGEN, Hilden, Germany), and bisulfite conversion was performed with the EZ DNA Methylation-Direct Kit (Zymo Research, Irvine, CA, USA). DNA concentrations were measured using a NanoDrop One spectrophotometer (Thermo Fisher Scientific, Waltham, MA, USA), and 2 ng of bisulfite-converted DNA was used per 10 µL of polymerase chain reaction (PCR) mixture.

Quantitative methylation-specific PCR was performed using a LightCycler^®^ 480 System II (Roche Diagnostics, Basel, Switzerland) with probe-based (TaqMan) assays. Primers and probes for *p16* promoter [20], *p16* exon 2 [22], *CDH13* [20,22], *RASSF1A* [20,22], and *ACTB* (β-actin) [18] were designed based on previously published sequences (Table S1). The final primer and probe concentrations were 600 and 200 nM, respectively. PCR mixtures were prepared using Platinum™ Taq DNA Polymerase, DNA-free (Invitrogen, Carlsbad, CA, USA) in a total volume of 10 µL. Cycling conditions comprised an initial denaturation at 95 °C for 1 min, followed by 50 cycles of denaturation at 95 °C for 15 s and annealing/extension at 60 °C (63 °C for *CDH13*) for 1 min.

Standard curves were generated using a bisulfite converted Universal Methylated Human DNA Standard (Zymo Research, Irvine, CA, USA) over a range of 4 to 1/64 ng (Figure S1). *ACTB* was used as the reference gene to normalize input DNA. The primers and probes targeting *ACTB* were designed to be unaffected by bisulfite-induced sequence changes. The percentage of methylated reference (PMR) was calculated as follows: PMR (%) = (amount of methylated target DNA estimated from the standard curve)/(amount of *ACTB* DNA estimated from the standard curve). PMR values below the detection limit of the standard curve were set to zero, whereas values exceeding 100% were capped at 100% to account for PCR variability.

### 2.4. Immunohistochemistry

Immunohistochemical staining was conducted using a BOND-III automated immunostainer (Leica Biosystems, Nussloch, Germany) and the BOND Polymer Refine Detection Kit (Leica Biosystems, Nussloch, Germany; DS9800) following the manufacturer’s protocol, with optimized conditions for each antibody against p16, CDH13, and RASSF1A. Primary antibodies were diluted in Dako REAL Antibody Diluent (Agilent Technologies, Santa Clara, CA, USA) as follows: p16: CINtec^®^ p16 Histology (Roche Diagnostics, Basel, Switzerland; 705-4713) was diluted to 1:8. Epitope retrieval was performed using BOND Epitope Retrieval Solution 2 (Leica Biosystems, Nussloch, Germany) for 20 min. CDH13: Goat polyclonal anti-CDH13 antibody (R&D Systems, Minneapolis, MN, USA; AF3264) was used at a 1:200 dilution. Epitope retrieval was performed for 40 min using BOND Epitope Retrieval Solution 1 (Leica Biosystems, Nussloch, Germany). The samples were incubated with the primary antibody for 60 min, followed by signal amplification using mouse anti-goat Immunoglobulin G-horseradish peroxidase (Santa Cruz Biotechnology, Dallas, TX, USA) diluted 1:100 in phosphate-buffered saline for 15 min. RASSF1A: Rabbit polyclonal anti-RASSF1A antibody (Sigma-Aldrich, St. Louis, MO, USA; HPA040735) was used at 1:400 dilution. Epitope retrieval was conducted using BOND Epitope Retrieval Solution 1 for 40 min. Samples were incubated with the primary antibodies for 60 min, followed by a 15-min signal amplification using Rabbit LINKER (Leica Biosystems, Nussloch, Germany).

To evaluate protein expression in tumors, 10 fields at ×200 magnification were selected to ensure coverage of the tumor area. Only tumor cells with unequivocal staining were counted in each field. Positive expression was defined as staining observed in ≥50% of tumor cells. Given that immunohistochemistry in this study was intended to characterize the predominant tumor phenotype and thereby aid the interpretation of methylation findings, a predefined threshold was selected to reflect the major clone rather than focal staining heterogeneity. This threshold was applied uniformly across all three markers to reduce inter-observer variability. CDH13 and RASSF1A staining was cytoplasmic, whereas p16 is expressed in the nucleus and cytoplasm; consistent with previous studies [28], only nuclear p16 staining was assessed. Evaluation was based exclusively on nuclear staining to minimize variability, as it is more consistently observed and widely reported for p16. Slides were evaluated by two observers blinded to clinical information and methylation results, and the final classification was assigned by consensus.

### 2.5. Statistical Analysis

All statistical analyses were performed using SPSS software version 28.0.1.0 (IBM Corp., Armonk, NY, USA); however, Fisher’s exact tests for three-group comparisons were conducted in R version 4.5.1 (R Foundation for Statistical Computing, Vienna, Austria). A two-sided *p*-value of <0.05 was considered significant.

Clinicopathological features of patients with LUSC across background lung diseases (IPF, PE, and SRIF) were compared using the Kruskal–Wallis test with Dunn’s post hoc test and Bonferroni correction for non-normally distributed continuous variables (age), one-way analysis of variance followed by Tukey’s post hoc test for normally distributed continuous variables (pack-years of smoking), and Fisher’s exact test for categorical variables.

DNA methylation levels among the background disease groups (IPF, PE, and SRIF) within each tissue region (T, A, and D) were compared using the Kruskal–Wallis test, followed by Dunn–Bonferroni post hoc comparisons. Within each disease group, differences in DNA methylation levels between tissue regions (T, A, and D) were assessed using the Wilcoxon signed-rank test with Bonferroni-adjusted *p*-values.

IHC levels across background disease groups (IPF, PE, and SRIF) were compared using Fisher’s exact test.

## 3. Results

### 3.1. Clinicopathological Features

No significant differences were observed among the IPF, PE, and SRIF groups with respect to age, sex, smoking status, tumor location, pathological subtype, or TNM classification (Table 1). LUSC arising in the setting of IPF tended to be located more frequently in the lower lobes, whereas LUSC associated with PE was more commonly observed in the upper lobes. These tendencies were consistent with the characteristic regional distributions of IPF and PE, respectively.

### 3.2. DNA Methylation Analysis

**Figure 2:**
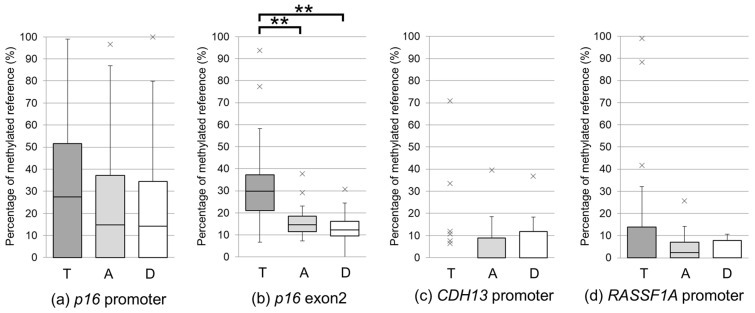
Methylation levels in tumor, adjacent, and distant tissues in background lung diseases. Methylation levels of *p16* promoter (**a**), *p16* exon 2 (**b**), *CDH13* promoter (**c**), and *RASSF1A* promoter (**d**) assessed in patients with LUSC (*n* = 25). Data are presented as box and whisker plots. Boxes represent tissue categories: tumor (T, dark gray), adjacent (A, light gray), and distant (D, white) lung tissues. Statistical significance was defined as a *p*-value of < 0.05 (* *p* < 0.05, ** *p* < 0.01). LUSC, lung squamous cell carcinoma.

In the overall cohort, *p16* exon 2 methylation levels were significantly higher in tumor tissues than in A and D regions (T > A and T > D; *p* < 0.001; Figure 2, Table S2).

**Figure 3:**
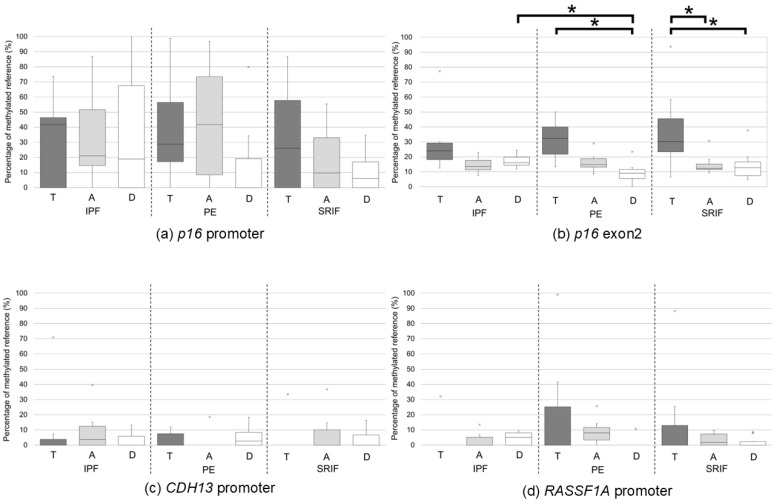
Methylation levels in patients with LUSC with IPF, PE, or SRIF according to sampling regions. Methylation levels of *p16* promoter (**a**), *p16* exon 2 (**b**), *CDH13* promoter (**c**), and *RASSF1A* promoter (**d**) assessed in patients with LUSC with IPF (*n* = 7), PE (*n* = 8), and SRIF (*n* = 10). Data are presented as box and whisker plots. Boxes represent background lung disease categories: tumor (T, dark gray), adjacent (A, light gray), and distant (D, white). Statistical significance was defined as a *p*-value of <0.05 (* *p* < 0.05, ** *p* < 0.01). LUSC, lung squamous cell carcinoma; IPF, idiopathic pulmonary fibrosis; PE, pulmonary emphysema; SRIF, smoking-related interstitial fibrosis.

The methylation levels in T, A, and D lung tissues from patients with IPF, PE, and SRIF are summarized in Figure 3, Figure S2 and Table S2. No significant differences were observed in the promoter methylation of *p16*, *CDH13*, and *RASSF1A* among disease groups or tissue regions. Within-group comparisons across tissue regions (T, A, and D) revealed distinct patterns according to the underlying lung disease. In patients with IPF, *p16* exon 2 methylation levels did not differ significantly among T, A, and D tissues. In contrast, patients with PE exhibited significantly higher methylation levels in T compared with D tissues (*p* = 0.035), while A tissues showed a trend toward higher methylation compared with D tissues, although this difference did not reach statistical significance (*p* = 0.052). In patients with SRIF, *p16* exon 2 methylation levels were significantly higher in T tissues than in both A (*p* = 0.028) and D (*p* = 0.015) tissues. Within-region comparisons across background disease types (IPF, PE, and SRIF) revealed that, in the D region, *p16* exon 2 methylation levels were significantly higher in the IPF group than in the PE group (*p* = 0.024). No other significant differences were observed among disease groups in any tissue region.

When tumor tissues were compared with combined non-tumorous samples (A + D), significant differences in *p16* exon 2 methylation were observed within each disease group (IPF, PE, and SRIF), whereas no significant differences were detected in promoter methylation of *p16*, *CDH13*, or *RASSF1A* (Table S3, Figure S3). Notably, the *p16* promoter methylation levels were significantly higher in patients with stage II–III LUSC than those with stage I disease (*p* = 0.048) (Table 2).

### 3.3. Immunohistochemistry

**Figure 4:**
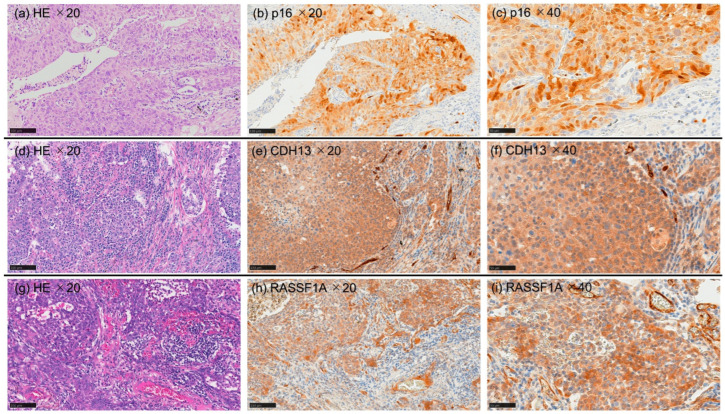
Representative immunohistochemical staining of p16, CDH13, and RASSF1A in tumor regions of LUSC. For each marker, a representative ×20 immunohistochemical (IHC) image is shown alongside the corresponding ×20 hematoxylin and eosin (HE) image and the corresponding enlarged ×40 IHC image (*p16*: (**a**–**c**), *CDH13*: (**d**–**f**), and *RASSF1A*: (**g**–**i**)). *CDH13* and *RASSF1A* were detected predominantly in the cytoplasm, whereas *p16* was expressed in the nucleus and cytoplasm. Panels (**a**,**b**,**d**,**e**,**g**,**h**) correspond to ×20 objective magnification fields (scale bars = 100 µm), and panels (**c**,**f**,**i**) correspond to ×40 objective magnification fields (scale bars = 50 µm). LUSC, lung squamous cell carcinoma.

**Figure 5:**
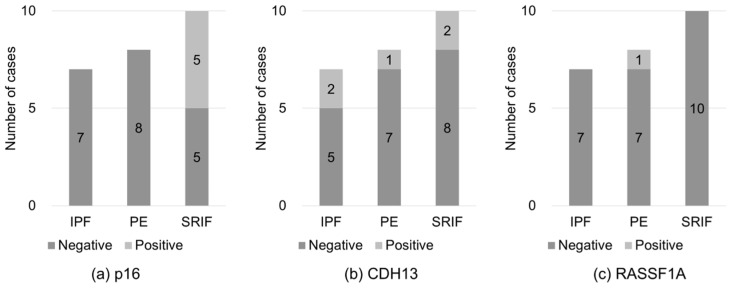
Immunohistochemical staining of p16, CDH13, and RASSF1A in tumor tissues. Immunohistochemical expression of p16 (**a**), CDH13 (**b**), and RASSF1A (**c**) in the tumor regions of patients with LUSC with IPF (*n* = 7), PE (*n* = 8), and SRIF (*n* = 10). Positivity was defined as ≥50% of the tumor cells exhibiting definitive nuclear staining for p16 or cytoplasmic staining for CDH13 and RASSF1A. Each bar represents the proportion of patients with positive (light gray) and negative (dark gray) staining, with the corresponding number of patients indicated within each bar. IPF, idiopathic pulmonary fibrosis; PE, pulmonary emphysema; SRIF, smoking-related interstitial fibrosis; LUSC, lung squamous cell carcinoma.

Representative immunohistochemical staining patterns of p16, CDH13, and RASSF1A in tumor and background lung tissues are shown in Figure 4. The positivity rates in tumor tissues are summarized in Table 3 and Figure 5. Based on the criteria described in Section 2, p16-positive staining was observed in five of ten patients with SRIF (50.0%), whereas no p16 positivity was detected in patients with IPF or PE, representing a statistically significant difference among disease groups (*p* = 0.041).

For CDH13, positive staining was detected in two of seven patients with IPF (28.6%), one of eight patients with PE (12.5%), and two of ten patients with SRIF (20.0%); however, these differences were not statistically significant. Similarly, RASSF1A positivity was observed in one of eight patients with PE (12.5%), whereas no positive staining was detected in patients with IPF or SRIF, with no significant differences among groups.

## 4. Discussion

In this study, we demonstrated frequent hypermethylation of *p16* exon 2 in tumor tissues in patients with LUSC arising in IPF, PE, and SRIF (Figure 3, Table S2). Consistent with this observation, *p16* exon 2 methylation levels were significantly higher in tumor tissues than in adjacent and distant non-tumorous lung tissues in the overall cohort (T > A and T > D) (Figure 2). This difference remained significant when tumor tissues were compared with the combined non-tumorous regions within each disease group (T > A and D; Table S3, Figure S3), supporting the notion that *p16* exon 2 hypermethylation is a tumor-associated molecular event in LUSC.

However, stratified analyses by underlying lung disease revealed distinct patterns. In patients with PE and SRIF, *p16* exon 2 methylation was significantly higher in tumor tissues than in adjacent and/or distant lung tissues, consistent with a tumor-restricted epigenetic alteration. In contrast, in patients with IPF, no statistically significant differences were observed between tumor tissues and non-tumorous lung tissues, and elevated *p16* exon 2 methylation was also detected in distant non-tumorous regions. These findings suggest that while *p16* exon 2 hypermethylation is a common molecular alteration in LUSC, its spatial distribution across tumor and background lung tissues differs according to the underlying lung disease, potentially reflecting disease-specific mechanisms of carcinogenesis.

In D lung regions, *p16* exon 2 methylation was significantly higher in patients with IPF than in those with PE, whereas no significant difference was observed between IPF and SRIF. Given that adjacent and distant regions were defined by anatomical distance, the D region should not be interpreted as a biologically unaffected baseline; rather, it represents a distant non-tumorous region that may still reflect disease-related and FC-related molecular alterations and cellular interactions. Accordingly, the pattern of *p16* exon 2 methylation in D regions suggests that the lungs affected by IPF may harbor more extensive molecular alterations consistent with FC, while carcinogenic changes in PE appear to be more localized. Given the high incidence of lung cancer in patients with IPF [7,8], the presence of elevated *p16* exon 2 methylation in distant non-tumorous lung tissue supports the concept of an expanded precancerous field in IPF. In this context, moderate *p16* exon 2 methylation may reflect a precancerous state, whereas further increases likely accompany malignant transformation or tumor progression.

To date, *p16* exon 2 methylation has not been established as a diagnostic marker in LUSC [41], and its status has not been investigated in LUSC with comorbid IPF, PE, or SRIF. However, *p16* exon 2 hypermethylation has been reported in several malignancies, including LUAC [22,23], colorectal [42], bladder [43], esophageal [27], and breast cancers [44], and has been associated with tumor progression and poor prognosis in some of these contexts [27,43]. Moreover, progressive increases in *p16* exon 2 methylation normal tissue to precancerous lesions and carcinoma have been described in LUAC [22] and breast cancer [44]. Although no direct association between *p16* exon 2 methylation and transcriptional silencing [45] or protein expression [27] has been demonstrated, our findings suggest that this epigenetic alteration may serve as a marker of molecular changes associated lung carcinogenesis, particularly within a FC framework.

In contrast, *p16* promoter methylation did not differ significantly among background lung disease groups or between tumor and non-tumorous tissues. Although this appears inconsistent with reports describing frequent *p16* promoter hypermethylation in lung cancer [18,19,20,21], a previous study had demonstrated comparable methylation levels in tumor and adjacent histologically normal lung tissues [23], suggesting that promoter methylation may represent a smoking-related or FC-associated epigenetic change rather than a tumor-tissue-specific event. Notably, *p16* promoter methylation was the only clinicopathological factor associated with tumor stage, with higher methylation levels observed in stage II-III LUSC than in stage I disease. This observation is consistent with prior reports linking *p16* promoter methylation to metastasis and poor prognosis in NSCLC across disease stages [46,47,48], and may reflect disease progression rather than tumor initiation.

No clear associations were observed between promoter methylation of *CDH13* and *RASSF1A* and LUSC across the assessed background lung diseases. This finding aligns with previous reports indicating that promoter methylation of these genes is more prevalent in LUAC than in LUSC [49,50]. These results suggest that the carcinogenic pathways highlighted in the present study may proceed independently of *CDH13* or *RASSF1A* methylation, although validation in larger cohorts and with broader gene panels is warranted.

At the protein level, p16 expression was detected in tumor tissues from patients with SRIF but not in those with IPF or PE. Consistent with previous observations [2], tumors in IPF and PE arose within or adjacent to fibrotic or emphysematous regions, whereas tumors in SRIF frequently developed outside the fibrotic lesions. These findings suggest that the molecular characteristics and carcinogenic mechanisms of LUSC in SRIF may differ from those in IPF or PE. Given that reduced p16 expression was correlated with poor prognosis in NSCLC [28,29,30], the absence of p16 expression in IPF- or PE-associated LUSC may reflect a more aggressive tumor phenotype, potentially contributing to the poor prognosis. This possibility may be particularly relevant in IPF-associated LUSC, consistent with the unfavorable clinical outcomes reported for lung cancers arising in this background. Such an aggressive tumor phenotype may also underlie the methylation patterns observed in the present study, as well as the associated FC pattern.

Several limitations should be acknowledged. First, heterogeneity in cellular composition between tumor-adjacent and tumor-distant lung tissues may have influenced methylation and immunohistochemical findings, as background lung tissues comprise variable proportions of epithelial and stromal cell [4]. Furthermore, recent integrative analyses have identified *NSD3* amplification as a critical genetic driver of LUSC tumorigenesis, associated with a non-inflamed, immune-desert phenotype and inferior immunotherapy outcomes, highlighting the need for future studies integrating immune cell composition with molecular alterations and tumor behavior [51]. Nevertheless, unlike prior studies [20,22,23], we applied predefined criteria for sampling non-tumorous regions, enabling a more controlled evaluation of spatial epigenetic alteration. Second, the limited sample size and lack of longitudinal follow-up precluded assessment of associations among methylation status, tumor progression, and patient outcomes. Moreover, the small sample size may have reduced the ability to detect true differences; for example, although *p16* exon 2 methylation in D regions was higher in IPF than in SRIF, this difference may not have reached statistical significance because of limited power. Larger, longitudinal studies will be necessary to determine whether *p16* exon 2 methylation can serve as a biomarker for malignancy risk or recurrence. Such studies may also help clarify whether complex, elusive mechanisms underlying *p16* aberrations contribute to LUSC carcinogenesis. This possibility is supported by the distinct spatial pattern of *p16* exon 2 methylation, the stage-related increase in *p16* promoter methylation, and the discordant *p16* immunohistochemical expression observed across background lung diseases.

It should be noted that lung tissues distant from the tumor may still harbor early molecular alterations related to FC. Therefore, the molecular changes observed in the D region may reflect disease-related alterations associated with the underlying lung condition and early FC–related effects. Conversely, the alterations observed in A regions may partly reflect tumor-associated field effects. Further studies using non-cancer controls will be required to clearly distinguish disease-related changes from tumor-associated field effects.

In conclusion, this study demonstrates that *p16* exon 2 methylation is a common molecular feature of LUSC, with distinct spatial and disease-related patterns depending on the background lung disease. While tumor-restricted hypermethylation predominated in PE and SRIF, IPF exhibited elevated methylation extending into distant lung tissue, consistent with an expanded FC effect. This finding underscores the heterogeneity of carcinogenic pathways in LUSC arising from different chronic lung diseases.

## Figures and Tables

**Table 1:** Clinicopathological characteristics of patients with lung squamous cell carcinoma.

Clinicopathological Parameters	IPF (*n* = 7)	PE (*n* = 8)	SRIF (*n* = 10)	*p*-Value
Age (years) [Median (IQR)]	73.0 (18.0)	70.5 (8.5)	76.0 (6.5)	NS
Sex [Male/Female]	6/1	8/0	9/1	NS
Smoking status (pack-years) [Mean (SD)]	37.9 (13.9)	50.3 (9.4)	61.9 (28.7)	NS
Location of tumor [upper lobe/middle lobe/lower lobe]	2/0/5	6/1/1	6/0/4	NS
Pathological Subtype [Keratinizing/non-keratinizing]	6/1	8/0	9/1	NS
Tumor stage				NS
T1	1	4	1	
T2	4	2	8	
T3	2	1	1	
T4	0	1	0	
Nodal stage				NS
N0	4	6	8	
N1	1	1	2	
N2	2	1	0	
Metastatic status				NS
M0	7	8	10	
M1	0	0	0	
Clinicopathological TNM stage				NS
I	4	3	8	
II	1	3	1	
III	2	2	1	

TNM factor and TNM stage were statistically compared using cutoff values of 0 vs. ≥1, <2 vs. ≥2 and <3 vs. ≥3, yielding the same results. IPF, idiopathic pulmonary fibrosis; PE, pulmonary emphysema; SRIF, smoking-related interstitial fibrosis; NS, not significant (*p* > 0.05).

**Table 2:** Comparison of DNA methylation levels in tumor tissues between stage I and stage II-III lung squamous cell carcinoma.

Gene	Stage I (*n* = 15)		Stage II–III (*n* = 10)		*p*-Value
	Median PMR (%)	IQR (%)	Median PMR (%)	IQR (%)	
*p16* promoter	0.0	41.8	49.3	47.2	0.048
*p16* exon 2	24.9	29.0	30.2	20.4	NS
*CDH13* promoter	0.0	7.6	0.0	0.0	NS
*RASSF1A* promoter	0.0	19.7	0.0	10.4	NS

PMR, percentage of methylated reference; NS, not significant (*p* > 0.05).

**Table 3:** Immunohistochemical expression of p16, CDH13, and RASSF1A proteins in tumor tissues.

Protein	Background Lung Disease	Positive/Total	*p*-Value
p16	IPF	0/7	*p* = 0.006
	PE	0/8	
	SRIF	5/10	
CDH13	IPF	2/7	NS
	PE	1/8	
	SRIF	2/10	
RASSF1A	IPF	0/7	NS
	PE	1/8	
	SRIF	0/10	

Immunohistochemical positivity rates of p16, CDH13, and RASSF1A in tumor tissues of patients with LUSC with IPF, PE, and SRIF. Positivity was defined as ≥50% of the tumor cells exhibiting definitive nuclear staining for p16 or cytoplasmic staining for CDH13 and RASSF1A. IPF, idiopathic pulmonary fibrosis; PE, pulmonary emphysema; SRIF, smoking-related interstitial fibrosis; LUSC, lung squamous cell carcinoma; NS, not significant (*p* > 0.05).

## Data Availability

The data presented in this study are available on request from the corresponding author.

## Supplementary Materials

The following supporting information can be downloaded at: https://www.mdpi.com/article/10.3390/curroncol33040187/s1, Table S1. primer and probe sequences used for quantitative methylation-specific polymerase chain reaction; Table S2. summary of DNA methylation analysis in tumor, adjacent, and distant lung tissues; Table S3. methylation levels of *p16*, *CDH13*, and *RASSF1A* in tumor and non-tumorous lung regions; Figure S1. Standard curves for the qMSP assay; Figure S2. Methylation levels in tumor, adjacent, and distant tissues according to background lung diseases; Figure S3. Methylation levels of *p16*, *CDH13*, and *RASSF1A* in tumor and non-tumorous lung regions.

## Abbreviations

The following abbreviations are used in this manuscript:

FC	Field cancerization
LUAC	Lung adenocarcinoma
NSCLC	Non-small cell lung cancer
IPF	Idiopathic pulmonary fibrosis
PE	Pulmonary emphysema
SRIF	Smoking-related interstitial fibrosis
LUSC	Lung squamous cell carcinoma
ECM	Extracellular matrix
EMT	Epithelial–mesenchymal transition
FFPE	Formalin-fixed paraffin-embedded
PMR	Percentage of methylated reference
T	Tumor region
A	Tumor-adjacent region
D	Tumor-distant region
