# Supplementary material for: CDKN2A/p16 Exon 2 Hypermethylation in Lung Squamous Cell Carcinoma Associated with Interstitial and Emphysematous Lung Diseases: A Comparative Analysis of Tumor, Adjacent and Distant Lung Tissues
Source: Curr Oncol. 2026 Mar 27;33(4):187. doi: 10.3390/curroncol33040187 (PMC13114730; doi:10.3390/curroncol33040187)
Supplement: Supplementary file 1 [file curroncol-33-00187-s001.zip › curroncol-4162880-supplementary.pdf]

Current Oncology

MDPI

Supplementary Materials

Table S1. Primer and probe sequences used for quantitative methylation-specific polymerase chain reaction

Genes	Forward primer	Probe	Reverse primer
p16 promoter	AACAACGCCCGCACCTCCT	ACCCGACCCCGAACCGCG	TGGAGTTTTCGGTT-GATTGGTT
p16 *exon 2*	GCGTTCGAGTGGCGGA	CAATTAAACTCCGCGCCG-TAAAACAACAA	CTCCCGAACAACGTCG-TACAC
CDH13 promoter	AATTTCGTTCGTTTTGTGCGT	AACGCAAAACGCGCCCGACA	CTACCCGTACCGAAC-GATCC
RASSF1A promoter	ATTGAGTTGCGGGAGTTGGT	CCCTTCCCAACGCGCCCA	ACACGCTCCAACCGAA-TACG
ACTB	TGGTGATGGAGGAGGTTTAG-TAAGT	ACCACCACCCAACACACAA-TAACAAACACA	AACCAATAAAAC-CTACTCCTCCCTTAA

Table S2. Summary of DNA methylation analysis in tumor, adjacent, and distant lung tissues

Gene	Background lung disease	Area type	Median PMR (%)	IQR (%)	*p*-value
p16 promoter	Total	T	27.4	51.6	[T vs. A] : NS
		A	14.9	37.3	[T vs. D] : NS
		D	14.2	34.3	[A vs. D] : NS
	IPF	T	41.8	46.4	
		A	21.1	37.0	[IPF vs. PE] in T: NS, in A: NS, in D: NS
		D	19.0	67.4	[IPF vs. SRIF] in T: NS, in A: NS, in D: NS
	PE	T	28.7	39.3	[PE vs. SRIF] in T: NS, in A: NS, in D: NS
		A	41.8	64.9	
		D	0.0	19.2	[T vs. A] in IPF: NS, in PE: NS, in SRIF: NS
	SRIF	T	24.9	48.2	[T vs. D] in IPF: NS, in PE: NS, in SRIF: NS
		A	0.0	15.8	[A vs. D] in IPF: NS, in PE: NS, in SRIF: NS
		D	16.8	32.9	
p16 exon2	Total	T	29.8	16.2	[T vs. A] : $p < 0.001$
		A	14.6	7.0	[T vs. D] : $p < 0.001$
		D	12.3	6.7	[A vs. D] : NS
	IPF	T	24.0	11.1	
		A	13.6	6.3	[IPF vs. PE] in T: NS, in A: NS, **in D[a]: 0.024**
		D	16.2[a]	5.5	[IPF vs. SRIF] in T: NS, in A: NS, in D: NS
	PE	T	32.5[c]	18.3	[PE vs. SRIF] in T: NS, in A: NS, in D: NS
		A	15.0	5.7	
		D	9.0[a,c]	6.2	[T vs. A] **in IPF: 0.054 (NS)**, in PE: NS, **in SRIF[b]: 0.028**
	SRIF	T	30.2[b,d]	22.1	[T vs. D] in IPF: NS, **in PE[c]: 0.035, in SRIF[d]: 0.015**
		A	14.6[b]	5.6	[A vs. D] in IPF: NS, **in PE: 0.052 (NS)**, in SRIF: NS
		D	12.0[d]	4.4	
CDH13 promoter	Total	T	0.0	0.0	[T vs. A] : NS
		A	0.0	8.9	[T vs. D] : NS
		D	0.0	11.8	[A vs. D] : NS
	IPF	T	0.0	3.8	

		A	3.6	12.4	[IPF vs. PE] in T: NS, in A: NS, in D: NS
		D	0.0	5.9	[IPF vs. SRIF] in T: NS, in A: NS, in D: NS
	PE	T	0.0	7.5	[PE vs. SRIF] in T: NS, in A: NS, in D: NS
		A	0.0	0.0	
		D	2.7	8.4	[T vs. A] in IPF: NS, in PE: NS, in SRIF: NS
	SRIF	T	0.0	0.0	[T vs. D] in IPF: NS, in PE: NS, in SRIF: NS
		A	0.0	6.7	[A vs. D] in IPF: NS, in PE: NS, in SRIF: NS
		D	0.0	10.1	
RASSF1A promoter	Total	T	0.0	13.8	[T vs. A] : NS
		A	2.4	7.0	[T vs. D] : NS
		D	0.0	7.7	[A vs. D] : NS
	IPF	T	0.0	0.0	
		A	0.0	5.3	[IPF vs. PE] in T: NS, in A: NS, in D: NS
		D	5.2	8.2	[IPF vs. SRIF] in T: NS, in A: NS, in D: NS
	PE	T	0.0	25.2	[PE vs. SRIF] in T: NS, in A: NS, in D: NS
		A	8.1	8.3	
		D	0.0	0.0	[T vs A] in IPF: NS, in PE: NS, in SRIF: NS
	SRIF	T	0.0	12.3	[T vs. D] in IPF: NS, in PE: NS, in SRIF: NS
		A	0.0	2.5	[A vs. D] in IPF: NS, in PE: NS, in SRIF: NS
		D	6.2	8.2	

PMR values for *p16* promoter, *p16* exon 2, *CDH13*, and *RASSF1A* in tumor (T), adjacent (A), and distant (D) lung regions among patients with LUSC with IPF, PE, and SRIF. PMR values were quantified using quantitative methylation-specific polymerase chain reaction. Statistical comparisons are shown in brackets, indicating the two groups compared, followed by the disease group or tissue regions after "in," and the corresponding p-value after the colon ":" (e.g., [T vs A] in IPF: NS). PMR, Percentage of methylated reference; IPE, idiopathic pulmonary fibrosis; PE, pulmonary emphysema; SRIF, smoking-related interstitial fibrosis; LUSC, lung squamous cell carcinoma; NS, non-significant

Table S3. Methylation levels of *p16*, *CDH13*, and *RASSF1A* in tumor and non-tumorous lung regions

Gene	background lung disease	Area type	Median PMR (%)	IQR (%)	*p*-value
p16 promoter	IPF	T	41.8	46.4	IPF: NS
		A+D	20.0	62.2	
	PE	T	28.7	39.3	PE: NS
		A+D	12.8	52.5	
	SRIF	T	26.2	57.8	SRIF: NS
		A+D	6.2	22.8	
p16 ex2 exon 2	IPF	T	24.0	11.1	IPF: 0.028
		A+D	14.8	6.8	
	PE	T	32.5	18.3	PE: 0.002
		A+D	12.4	7.7	
	SRIF	T	30.2	22.1	SRIF: 0.002
		A+D	12.3	5.1	
CDH13 promoter	IPF	T	0.0	3.8	IPF: NS
		A+D	0.0	11.3	
	PE	T	0.0	7.5	PE: NS
		A+D	0.0	5.8	
	SRIF	T	0.0	0.0	SRIF: NS
		A+D	0.0	10.0	

RASSF1A promoter	IPF	T	0.0	0.0	IPF: NS
		A+D	1.8	7.3	
	PE	T	0.0	25.2	PE: NS
		A+D	0.0	9.5	
	SRIF	T	0.0	13.0	SRIF: NS
		A+D	0.0	6.6	

PMR values for *p16* promoter, *p16* exon 2, *CDH13*, and *RASSF1A* in tumor (T) and non-tumorous (A + D) lung regions among patients with LUSC with IPF, PE, and SRIF. PMR values were quantified using quantitative methylation-specific polymerase chain reaction. PMR, Percentage of methylated reference; IPE, idiopathic pulmonary fibrosis; PE, pulmonary emphysema; SRIF, smoking-related interstitial fibrosis; LUSC, lung squamous cell carcinoma; NS, non-significant

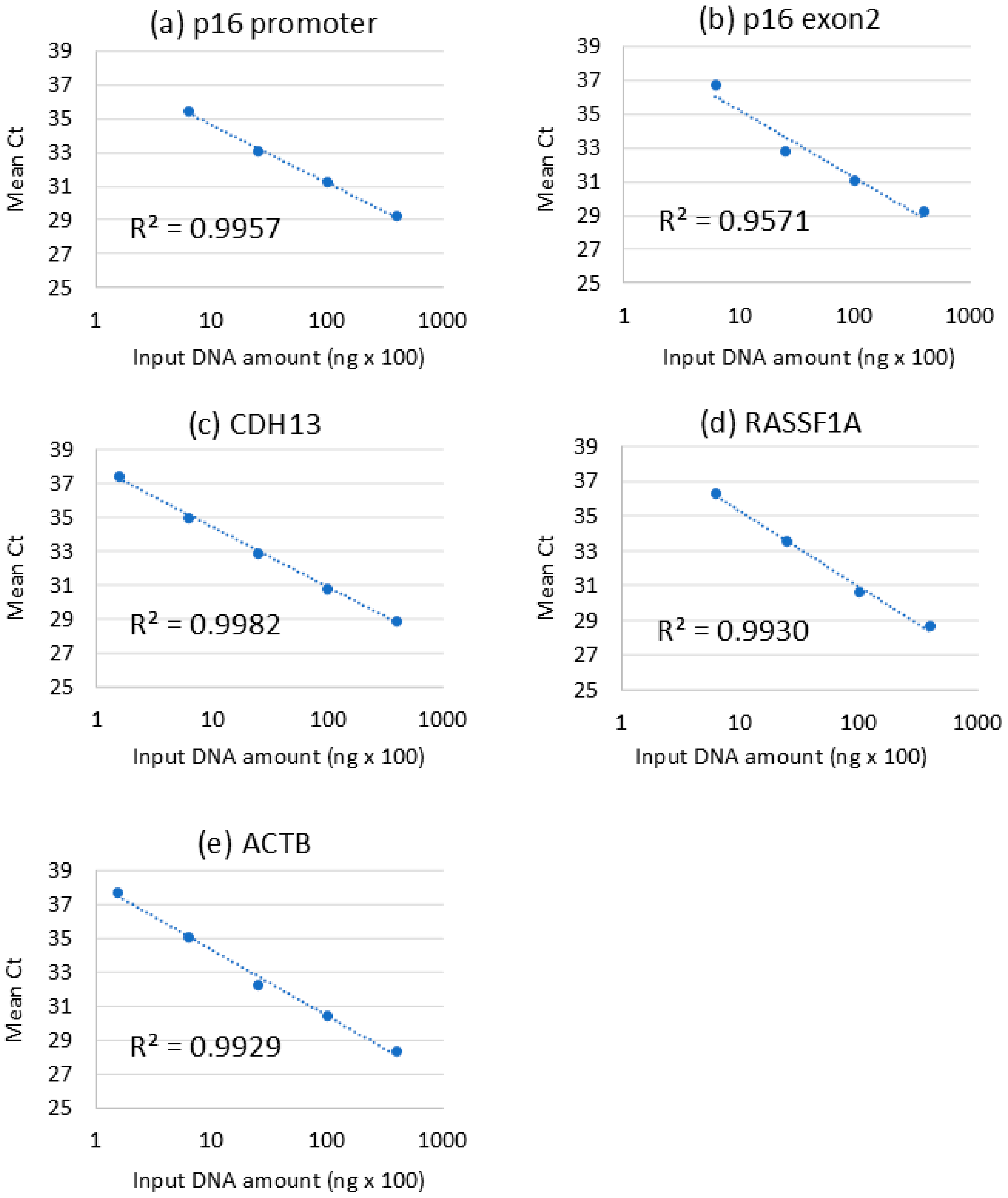

Figure S1. Standard curves for the qMSP assay. Standard curves for the *p16* promoter (**a**), *p16* exon 2 (**b**), *CDH13* promoter (**c**), *RASSF1A* promoter (**d**), and *ACTB* (**e**) were generated using a bisulfite converted Universal Methylated Human DNA Standard over a range of 4 to 1/64 ng. The x-axis represents the DNA input amount (ng × 100), and the y-axis represents the Ct value. Data points indicate the mean values of technical replicates. Linearity was confirmed over this range, and the corresponding coefficients of determination (R^2) are shown for each assay. No amplification was observed in the no-template controls.

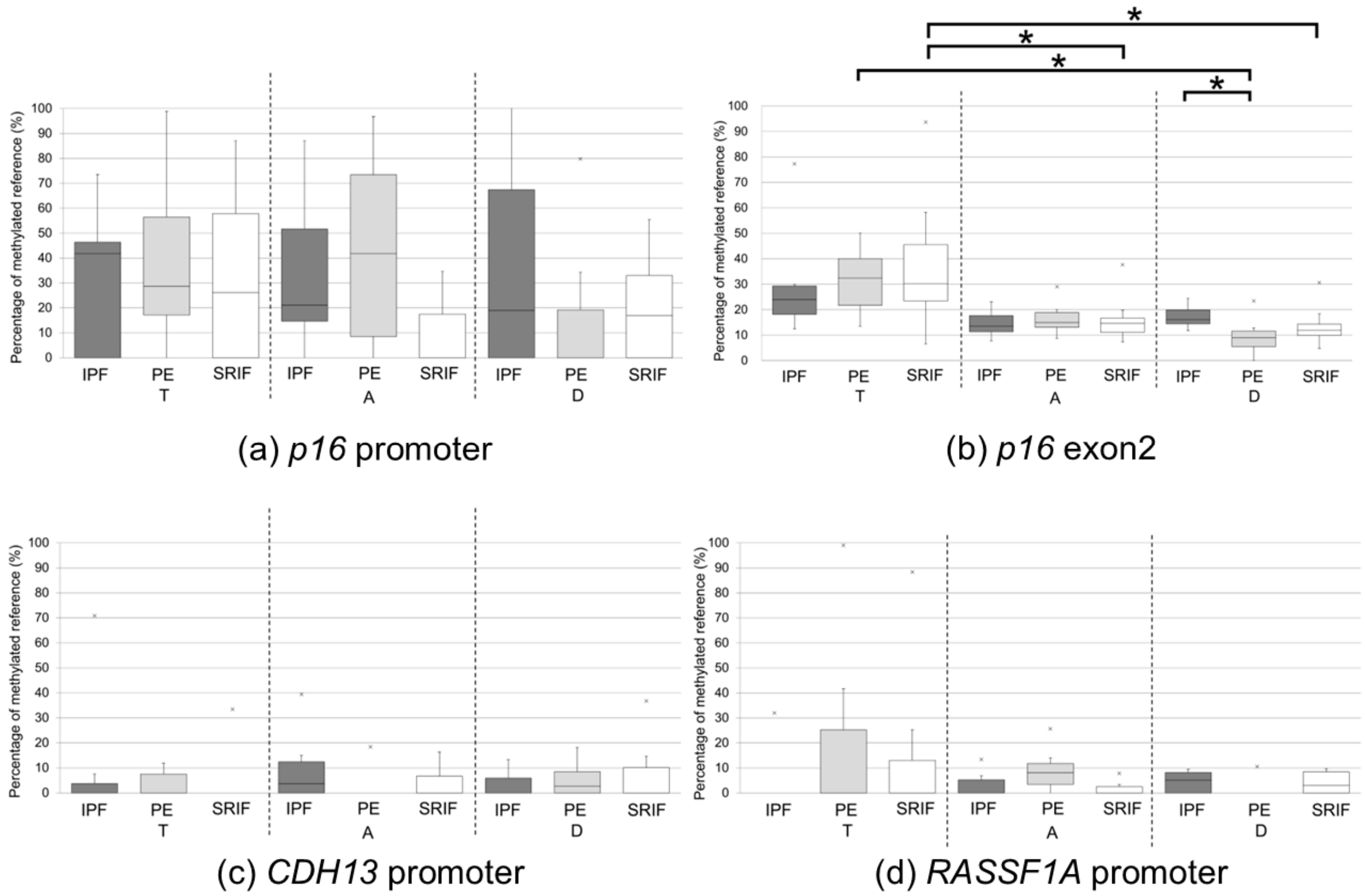

Figure S2. Methylation levels in tumor, adjacent, and distant tissues according to background lung diseases. Methylation levels of *p16* promoter (**a**), *p16* exon 2 (**b**), *CDH13* (**c**), and *RASSF1A* (**d**) assessed in tumor (T), adjacent (A), and distant (D) lung tissues of patients with LUSC with IPF (n = 7), PE (n = 8), and SRIF (n = 10). Boxes represent background lung disease categories. Data are presented as box and whisker plots. Statistical significance was defined as a p-value of < 0.05 (*$p < 0.05$, **$p < 0.01$). IPE, idiopathic pulmonary fibrosis; PE, pulmonary emphysema; SRIF, smoking-related interstitial fibrosis; LUSC, lung squamous cell carcinoma

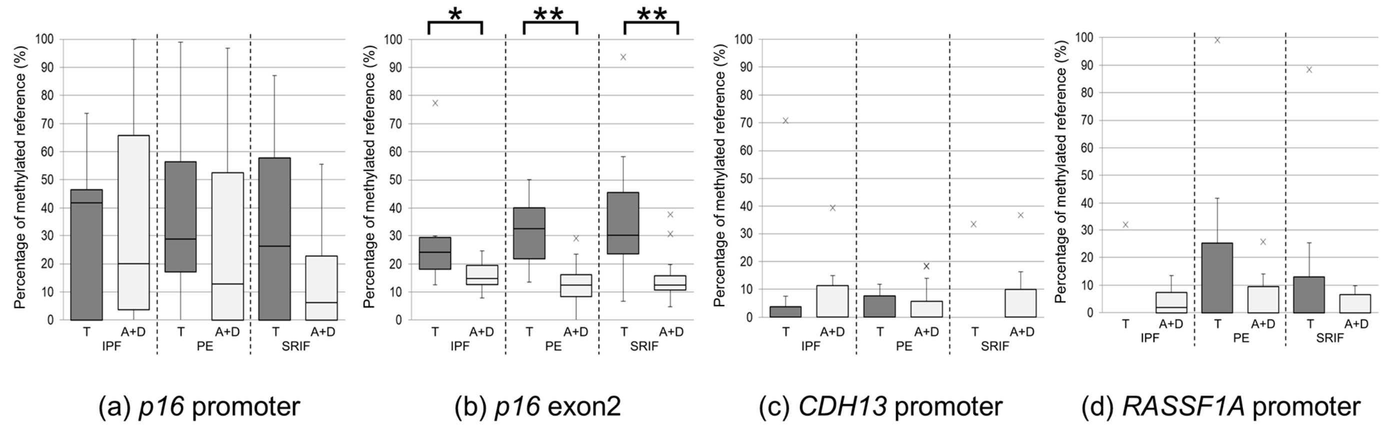

Figure S3. Methylation levels of *p16*, *CDH13*, and *RASSF1A* in tumor and non-tumorous lung regions. Percentage of methylated reference values for *p16* promoter (**a**), *p16* exon 2 (**b**), *CDH13* (**c**), and *RASSF1A* (**d**) in tumor (T, dark gray boxes) and non-tumorous (A + D, light gray boxes) lung regions were quantified using quantitative methylation-specific polymerase chain reaction. Statistical significance was defined as p-value of < 0.05 (*). IPE, idiopathic pulmonary fibrosis; PE, pulmonary emphysema; SRIF, smoking-related interstitial fibrosis
